# Effect of Phenolic Resin on Micropores Development in Carbon Foam with High Performance

**DOI:** 10.3390/ma12081213

**Published:** 2019-04-13

**Authors:** Alei Dang, Zhao Zhao, Chen Tang, Chenglin Fang, Siyuan Kong, Muhammad Khan, Tiehu Li, Tingkai Zhao, Hao Li

**Affiliations:** 1 State Key Laboratory of Solidification Processing, Shaanxi Engineering Laboratory for Graphene New Carbon Materials and Applications, School of Materials Science and Engineering, Northwestern Polytechnical University, Xi’an 710072, China; zhaozhao@mail.nwpu.edu.cn (Z.Z.); mecdoll@yahoo.com (C.T.); fangchenglin@mail.nwpu.edu.cn (C.F.); 15764256390@163.com (S.K.); mkhanchemistry@yahoo.com (M.K.); litiehu@nwpu.edu.cn (T.L.); ztk-xjtu@163.com (T.Z.); lihao@nwpu.edu.cn (H.L.); 2 NPU-NCP Joint International Research Center on Advanced Nanomaterials and Defects Engineering, Northwestern Polytechnical University, Xi’an 710072, China

**Keywords:** carbon foam, mechanical properties, phenolic resin, pyrolysis, modification

## Abstract

A novel high-performance carbon foam (CF) was fabricated through the addition of phenolic resin (PR) into a coal tar pitch (CTP) based precursor. The effects of mass fraction of a PR additive on the crystalline structures, morphologies, compressive strength (σ) and thermal conductivity (λ) of resultant CF material were investigated systematically. Characterization showed a strong dependence of CF’s performance from the composition and optical texture of the precursor, which were mainly depending on the polycondensation and polymerization reactions between PR and raw CTP. Comparing with the strength of pristine CF at 6.5 MPa, the σ of mCF-9 (13.1 MPa) was remarkably enhanced by 100.1%. However, the λ of mCF-9 substantially reduced to 0.9 m^−1^K^−1^ compared with 18.2 W m^−1^K^−1^ of pristine CF. Thus, this modification strategy to produce microporous CF materials from raw CTP provides a new protocol for the fabrication of high-performance carbon based materials.

## 1. Introduction

High-performance porous material, including metallic foam [1,2,3,4,5,6,7], metallic matrix syntactic foam [8], polymer foam [9,10,11,12], ceramic foam [13,14] and carbon foam [15,16] have attracted great attention during the tremendous growth of industrialization, owing to its excellent properties, such as lightweight, high specific surface area, low density and high strength. Hence, it has been considered as one major promising candidate for applications in environmental protection, vehicles, medicine and aviation industries [1,4,5,16]. Specially, carbon foam (CF) with a three dimensional reticular structure has gained much attention in the field of military and civilian utilization [16,17,18,19,20,21,22], owing to its excellent physical and chemical properties, such as high specific surface area and lightweight, extraordinary stability in harsh environments and tunable thermal and electrical conductivity [23]. CF having amorphous structure and thermally insulating properties was found for the first time by Ford. W. D in 1964 where the thermosetting organic polymer was being chosen for the precursor [24]. Later on, in order to achieve graphitic structure with thermally conductive CF, commercial mesophase pith [25] or modified coal tar pitch [26] were employed for the production of CF via simple thermal and high-pressure treatment. Nevertheless, fragile and poor mechanical properties of producing CF that mainly depended on the types of precursor and the pyrolysis conditions during the foaming process, restrict their further applications, such as structure adsorbents, electrode materials for supercapacitor and thermal energy storage devices [16,18,21,25].

To circumvent this issue, three methods have been developed in the last few decades to optimize the cell distribution and the mechanical performance of resulting CF [25], such as (1) regulate the foaming and carbonization conditions in the process of foaming; (2) modify the precursors by adding the cross-linking agent or modifier for optimizing the chemical composition and fluidity of precursor; (3) incorporate reinforcements into the precursor as graphite [26], coal powder [27], clay-montmorillonite [28], carbon nanotube [29,30], graphene [31], carbon filler [32] and ferrocene [33]. In contrast to the methods for adjusting the foaming condition and choosing reinforcements for the preparation of CF, the chemical modification was proposed due to its operability for modulating physical properties and chemical composition of precursor with low-costs [16]. Liu and coworkers obtained a high-mechanical performance CF derived from modified coal tar pitch (CTP) where the cinnamaldehyde and boric acid were employed for the modification of precursor [34]. It indicated that the microstructure and mechanical performance of prepared CF have been affected substantially by the composition and soft point of modified precursor. Tsynsarski et al. investigated the effect of the thermal oxidation treatment of precursor by inorganic acid (H_2_SO_4_ and HNO_3_) on the porosity development and properties of the resultant CF [35]. Zhu also reported that the compression strength of CF was augmented to some extent with the increasing ratios of coal to pitch [27]. 

In our previous work, we reported that the coking yield of modified CTP has been improved by 13% and soft point decreased by 25 °C with the variation of PR and catalyst content than that of raw CPT. It indicated that the chemical and physical properties of raw CTP were significantly influenced by the modification of PR, which can probably be used for improving the micropores’ structure and the performance of the resultant carbon foam. Hence, the aim of this work was to systematically investigate the effect of chemical composition of precursor CTP modified by the different PR additives on the variation of crystal structure, micropores’ distribution, bulking density, compressive strength (σ) and thermal conductivity (λ) of CF. This could provide an economical and facile method for the production of lightweight and high-strength CF.

## 2. Experimental

### 2.1. Preparation of Carbon Foam from Modified CTP

Due to the high viscosity of raw CTP, it is not appreciated as the precursor for achieving carbon foam directly [36]. Thus, in order to meet the foaming conditions, in this case, the PR was chosen as additive to reduce the viscosity of precursor in the presence of catalyst MBSA. The process of co-carbonization reaction of PR and raw CTP was mentioned in our previous work [37,38]. Here, the content of catalyst MBSA in the reaction system was invariant (1 wt%) for all samples. The modified CTP materials were noted as mCTP-1, mCTP-3, mCTP-5, mCTP-7 and mCTP-9 when the mass fraction of PR is equal to 1, 3, 5, 7 and 9 wt%, respectively. This showed that the chemical constitution and physical properties of CTP were largely influenced by the variation of PR. According to Isao Mochida’s work [39,40], the viscosity and crystal structure of precursor, which has a significant influence on the structure and micropores’ distribution of carbon foam [16], could be interpreted via an analysis of optical texture of precursor’s mesophase by using polarized light and a half-wave plate. Hence, to produce the mesophase of raw CTP and modified CTP materials, those precursors were further thermally treated as in reference [26].

For the production of carbon foam, the raw CTP and modified mCTP materials were typically ground to ~100 μm in size and placed into a high-pressure reactor vessel. Before the foaming process, the vessel was purged four times as expected through the charging and discharging cycle using nitrogen, which can be used to keep the oxygen free in the reaction system [18,34,38]. Here, the maximum pressure of nitrogen in vessel for each flushing is around 5 MPa. Then CF without carbonization was obtained when the temperature was increased to 450 °C and maintained for 5 h at 5 MPa for foaming. After that, the vessel was cooled down to room temperature where the nitrogen flows are always on before the sample is taken off. Subsequently, CF was synthesized in a quartz tube for carbonization at 950 °C for 4 h under the nitrogen atmosphere. The nitrogen supported the outer shape of the hybrid framework to avoid deformation and cracking of the CF material. After carbonization, a piece of porous CF was cleaned three times by ethanol and dried for 2 days in a vacuum oven. Finally, modified mCF-1, mCF-3, mCF-5, mCF-7 and mCF-9 samples were obtained from mCTP-1, mCTP-3, mCTP-5, mCTP-7 and mCTP-9 materials, respectively. 

### 2.2. Characterization

Extraction of toluene-soluble fraction (TS) and quinoline insoluble (QI) from the precursor markedly influenced the pore structure of foam-like materials [16,38]. Thus, the TS and QI of raw CTP, mC-1, mCTP-3, mCTP-5, mCTP-7 and mCTP-9 were analyzed in accordance to the STAS 8451/2 (1983) and STAS 8451/6 (1969), respectively. Chemical changes of pristine CF and mCF-5 samples were studied by FT-IR spectroscopy on a Bruker Tenser-27-FT-IR spectrometer (Karlsruhe, Germany). Measurements were performed in transmittance mode in the range of wave numbers from 4000 to 40 cm^−1^, and formed a KBr tablet weighing 300 mg with a sample to KBr weight ratio of 1:150. Moreover, to characterize the optical texture of mesophase, the epoxy resin was used to mount the resultant materials, and subsequently the mounted materials was separately polished by silicon carbide paper with different grades (such as 400, 600, 1200 grit). Furthermore, the sample polishing was finished by using Buehler MetaDi (Chicago, IL, USA) from 1 to 0.05 μm polishing suspension for 5 and 3 min respectively on a cloth pad. Later on, the optical texture of mesophases was characterized by OLYOPUMS-B061 (Shinjuku, Japan) reflected polarized-light microscopy and a half-wave plate.

The morphologies and microstructures of the as-prepared pristine CF and mCF series materials were characterized by scanning electron microscopy (SEM, FEI, Verios G4, Hillsboro, OR, USA) at an accelerating voltage of 5 kV. Meanwhile, Raman spectra of as-prepared pristine CF and mCF-5 was characterized using a Raman spectrometer coupled to an RM 2000 inverted confocal microscope (Alpha300R, Ulm, Germany) fitted with an Ar laser with a resolution of 2 cm^−1^ at a wavelength of 532 nm. Each spectrum was the result of four averages. Moreover, the phase identification was performed by X-ray diffraction (XRD, Shimadzu 7000, Kyoto, Japan) using nickel-filtered Cu *K*^α^ radiation. The scanning rate was 2° min^−1^ and the scanning angles ranged from 20° to 90° with a sampling width of 0.02°. Furthermore, the interlayer spacing (d_002_), which is calculated using the Brag equation (2d_002_sinθ = nλ, where θ, n and λ are the angle between the incident beam and the reflecting surface, diffraction series and the wavelength of incident light, respectively) was employed as an indicator for the degree of ordering.

The bulk density was evaluated by the weight and volume of pristine CF and mCF series materials. Here, the volume of samples was counted using the dimension of sample. The compression strength (σ) of samples (10 mm × 10 mm × 10 mm) was tested by two stainless platens with a crosshead speed of 0.5 mm/min using a universal testing machine (Instron 3382, Boston, MA, USA). Specifically, the σ was calculated by means of σ=F/A, where F and A are the load at yield and cross-section area, respectively. For the thermal property of resultant samples, the thermal conductivity (λ) was characterized by Netzsch LFA 457 conductivity tester (Selb, Germany) in the range of temperature between 25 °C–800 °C. Subsequently, the λ value (Wm^−1^K^−1^) can be calculated by the equation λ = ρCα, where ρ, C and α are the density (kg m^−3^), specific heat capacity (J kg^−1^K^−1^) and thermal diffusivity (m^2^ s^−1^) of samples. Finally, the results were obtained by the average of five tests.

## 3. Results and Discussions

For the production of carbon foam, the light components and thermally decomposed fractions in the precursors start to molten, saturate, nucleate, grow, merge and finally spill out after further heating [18,41]. Afterwards, with the reduction of light fractions, the precursor is further solidified and subsequently the structure of CF is fixed gradually. According to previous work, the microstructure and mechanical properties of CF were significantly affected by the viscosity, chemical composition and volume swelling of precursor [16]. Hence, in this case, the composition (such as TS and QI) of modified CTP, which is associated with the viscosity and volume swelling of the precursor, are employed to investigate the porosity distribution, mechanical and thermal performances of the resultant CF. 

### 3.1. Composition of Pristine CF and Modified mCF Materials

The characteristics of raw CTP and a series of as-prepared mCTP materials modified by different mass fraction of PR are summarized in Table 1. As showed in Table 1, compared to the raw CTP, the TS content of modified CTP materials decreases from 79.2 wt% to 75.8 wt% and corresponding QI increases from 3.9 wt% to 5.8 wt%, respectively, with the increase of mass fraction of PR from 0 wt% to 3 wt%. For the increased content of QI, it is ascribed to the formation of aromatic macromolecules resulting from the polycondensation and polymerization reactions of PR and raw CTP, which leads to the decrease of TS content. With a further increase in the mass fraction of PR to 5 wt%, the maximum values of QI content at 6.5 wt% is reached, indicating the active sites of the aliphatic and aromatic constituent in the precursor has reacted nearly with PR 5 wt%. On the contrary, when the mass fraction of PR reached to 9 wt%, the TS content of mCTP-9 increases to 76.4 wt% which is probably due to the excessive unreacted PR which exists in the precursor that is easily dissolved in such strong polar solvents such as toluene and quinoline, thus resulting in the increase of TS. 

**Figure 1:**
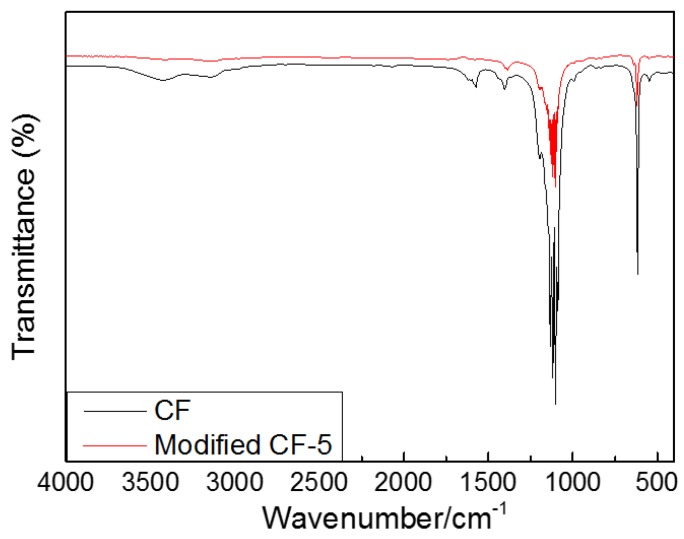
Comparison of FT-IR spectra of pristine CF and modified mCF-5.

Figure 1 illustrates the FT-IR absorption spectra of pristine CF and modified mCF-5 materials after co-carbonization at a temperature of 900 °C. From Figure 1, the FT-IR spectrum of the pristine CF displays two relatively weak absorption peaks in the range of 3300–3450 cm^−1^ and 3000–3100 cm^−1^, corresponding to O–H stretching vibration bands with hydrogen-bonded association and the aliphatic C–H stretching vibration bands, respectively. The band at 1573 cm^−1^ in the range of 1600–1500 cm^−1^ is assigned to the stretching of aromatic C=C groups, while the band in the region 1444–1377 cm^−1^ is due to the bending modes of saturated aliphatic hydrocarbons. However, those bands almost disappear in the FT-IR spectra of sample mCF-5, which indicates a reaction probably occurred after the modification of PR. The absorption peaks from 1300–1000 cm^−1^ and 700–900 cm^−1^ of sample mCF-5 are contributed from the C–O stretching vibrations of aromatic ether, phenols, phenolics and the out plane vibration of aromatic C–H, respectively. However, the much weaker intensity of absorption peaks in the FT-IR spectrum of sample m-CF is observed due to the consumption and loss of a great deal of volatiles and light compounds of the precursor during the modification process. 

### 3.2. Optical Structure of Pristine CF and Modified mCF Materials

**Figure 2:**
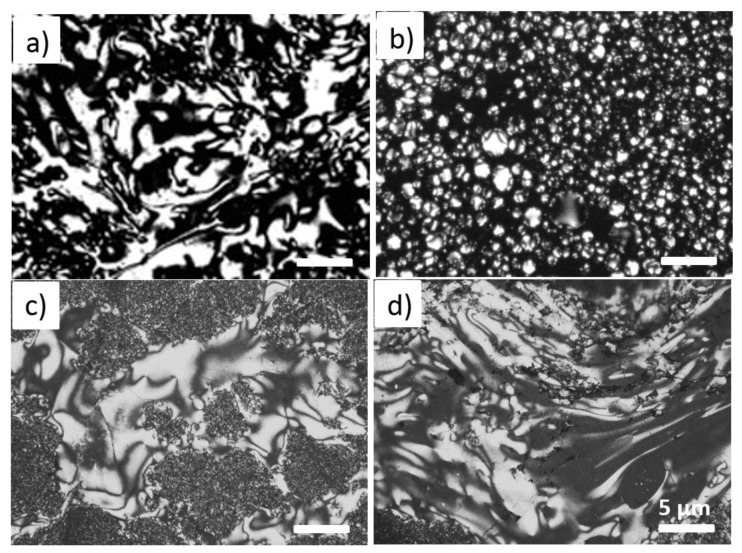
The polarized photomicrograph of mosophase of raw CTP (**a**), mCTP-1 (**b**), mCTP-5 (**c**) and m-CTP-9 (**d**) materials at 500 °C for 2 h, respectively. Scale bar are 5 μm for all.

The analysis of size variation of the isochromatic areas (also called macro-crystallinity areas) in the optical texture of the resultant materials that were obtained by the co-carbonization of additive and CTP can offer a discernment to the organic compounds in the additive with CTP [39,40]. Figure 2 shows the polarized photomicrographs of mesophase of raw CTP and modified CTP series materials, noted as mCTP-1, mCTP-5 and mCTP-9, where the mass fraction of PR additive for the modification of CTP are as same as 1 wt%, 5 wt% and 9 wt%, respectively. All the four samples are obtained by 2 h soaking time at 500 °C. From Figure 2a, a unit size in the range of 2–6 μm textured mosaic structure (noted as isochromatic area, which is from the birefringence phenomenon of the formed liquid crystal phase) is observed in the optical image of raw CTP’s mesophase, due to reorganization of anisotropic macromolecules in the liquid phase conditions. Here, the anisotropic molecules are mainly derived from recombination of radicals during the carbonization process.

With the increase of PR additives from 1 to 9 wt%, as shown in Figure 2b–d, the isochromatic areas in the optical micrographs of modified CTP series materials are much more different than that of raw CTP. When the amounts of PR equals to 1 wt%, a lot of small globules are formed in the optical images of mCTP-1 sample, indicating that a small amount of PR heavily impacts the liquefication and rearrangement of organic macromolecules, and corresponds to the formation of isochromatic areas. After a further increase of PR additive to 5 wt%, small domains in sizes of 10–50 μm with optically anisotropic structures are formed and no more obvious small globules appeared in the optical images. This confirms that the increase of PR amounts enlarged the isochromatic areas. In Figure 2d, the isochromatic area of sample mCF-9 almost occupied all the area. From the Isao Mochida’s work [39,40], the formed isochromatic area (mainly derived from mesophase or liquid crystal phase via the rearrangement of the anisotropic macromolecules) is usually associated with an extended temperature zone of fluidity in the carbonization system, which leads to a reduced viscosity and a higher fluidity. It will be beneficial to facilitate diffusion of larger molecules in precursors, and correspondingly to optimize the microstructure and micropores’ distribution of the resultant CF [34,38]. 

### 3.3. XRD and Raman Spectra of Pristine CF and Modified mCF Materials

**Figure 3:**
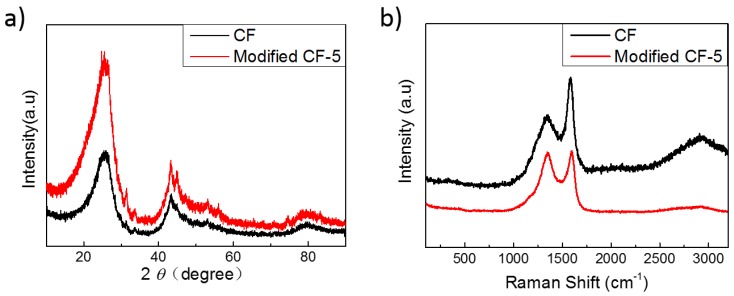
XRD patterns (**a**) and Raman spectra (**b**) of CF and modified CF-5.

To investigate the phase composition and crystal structure, XRD was employed for the characterization of materials [35]. Figure 3a displays the typical XRD spectra of pristine CF and sample mCF-5. As shown in Figure 3a, two broad diffraction peaks at around 25° and 43° are observed, which result from the (002) and (100) planes of micro-graphite respectively. In addition, according to the Bragg equation and Franklin formula [31,42], the interlayer spacing d_002_ of pristine CF and mCF-5 is 0.352 and 0.350 nm, respectively, indicating that the pristine CF and mCF-5 have an amorphous structure because of the larger d_002_ than graphite (0.3354 nm), as summarized in Table 2. Moreover, comparing with the graphitization degree of pristine CF (93.02%), the lower graphitization degree (69.8%) is obtained for mCF-5 due to the modification of CF by PR which led to the formation of macromolecular aromatic compounds and partial augment of the amorphous structure [42]. 

Furthermore, Figure 3b compares the Raman spectra of CF and mCF-5 samples in the 200–3500 cm^−1^ region at the laser emitting with 532 nm. In the Raman spectra, prominent two stronger peaks are observed at Raman shift 1357 (D band) and 1584 cm^−1^ (G band). Another peak at Raman shift around 2750 (G’ or 2D) is also found at the Raman spectra of pristine CF, which commonly exists in graphite materials [18]. Here, the D band is generally ascribed to the disordered carbons, whereas G band is assigned to the in-plane bond-stretching motion of the pairs of sp^2^ atoms [33,43]. From the Table 2, the *I*_D_/*I*_G_ of mCF-5 is much higher than that of pristine CF, where the *I*_D_/*I*_G_ ratio is equal to 0.99 and 0.51 for mCF-5 and pristine CF, due to the formation of partially disordered crystal structure and the defects of mCF-5. This result is consistent with the consequence of XRD pattern in Figure 3a. 

### 3.4. Morphologies of Pristine CF and Modified mCF Materials

**Figure 4:**
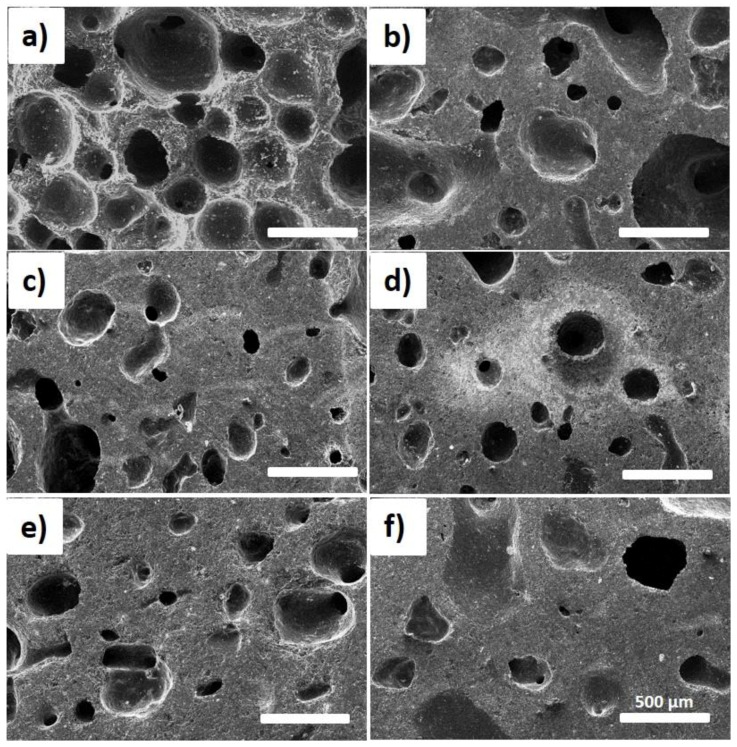
SEM images of pristine CF from raw CTP and as-prepared mCF materials from modified CTP. (**a**) pristine CF from CTP. (**b**–**f**) are sample mCF-1, mCF-3, mCF-5, mCF-7 and mCF-9 resulting from mCTP-1, mCTP-3, mCTP-5, mCTP-7 and mCTP-9, respectively. Scale bar is 500 μm for all.

**Figure 5:**
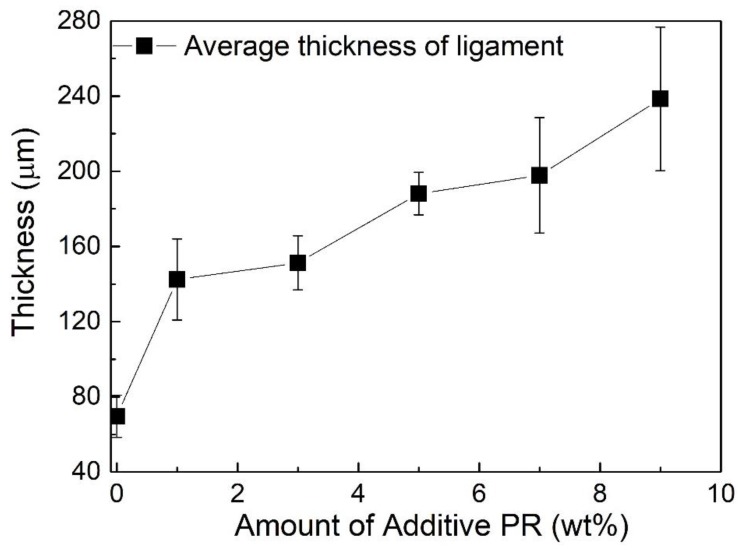
The thickness of as-prepared foams’ ligament varies with increase of amount of additive PR, where the thickness of ligament is 69.6 ± 11.2 μm for 0 wt% PR, 142.4 ± 21.6 μm for 1 wt%, 151.2 ± 14.4 μm for 3 wt%, 188.1 ± 11.3 μm for 5 wt%, 197.8 ± 30.7 μm for 7 wt% and 238.5 ± 38.2 μm for 9 wt%, respectively.

Since the physical performance and chemical composition of precursor have a significant impact on the cell shape, size and their distribution of resulting foams and corresponding mechanical properties, the PR is proposed to modify the foams’ precursor [16,26,27,34]. Figure 4 shows the SEM images of the cross-sectional morphologies of the pristine CF and mCF-1, mCF-3, mCF-5, mCF-7 and mCF-9, where the additive amount of PR is 1 wt%, 3 wt%, 5 wt%, 7 wt% and 9 wt%, respectively. In Figure 4a, the pristine CF exhibits a 3D foam architecture with interconnected micropores by narrow ligaments where the average thickness of ligaments is 69.4 ± 11.2 μm (as illustrated in Figure 5) and distribution of micropores are in the ranging of 200 μm to 500 μm. In Figure 4b,c, when the amount of PR is increased from 0 wt% to 3 wt%, mCF-1 and mCF-3 exhibit an inferior uniformity in size and relative thick ligament (142.4 ± 21.2 μm for mCF-1 and 151.2 ± 14.4 μm for mCF-3) of adjacent pores because of the produced aromatic macromolecules prevent the precursor domain’s nucleation, expanding, coalescence and reorientation. Finally, it leads to the formation of uneven and imperfect foams. 

Nevertheless, further increase the additive amounts of PR to 5 wt%, the uniformity of cell is dramatically approved and the range of the average size of a cell is dropped down from 150 μm to 300 μm due to the reduction of released volatile components and the formation of macromolecular aromatic compounds in the precursor, which are resulting from volatile component functional groups in the precursor that nearly reacted with PR (as discussed above), as shown in Figure 4d. Meanwhile, the average thickness of mCF-5 is reached to 188.1 ± 11.3 μm. Finally, when the PR amounts increased from 5 wt% to 9 wt% (197.8 ± 30.7 μm for 7 wt% and 238.5 ± 38.2 μm for 9 wt%), the inhomogeneous foam with more thick ligament between adjacent pores is observed, which is derived from the overflow of the excessive PR during the foaming process, as shown in Figure 5. 

### 3.5. Compression Strength of Pristine CF and Modified mCF Materials

The practical applications of CFs are limited substantially because of their fragility which leads to the structural damage of materials in service [16,33]. In this case, the compression strength (σ) of pristine CF and modified CF materials was investigated in detail. Table 3 summarizes the variation of σ and bulk density with different mass fraction of PR additives. As showed in Table 3, the strength of as-prepared mCF-1, mCF-5 and mCF-9 are 9.3, 12.8 and 13.1 MPa, respectively, which is enhanced by 43%, 96.9% and 100.1% than that of pristine CF (6.5 MPa), indicating that the addition of PR is beneficial to improve the mechanical performance of CF, and meanwhile the corresponded bulk density increased [34]. Table 3 shows that the compressive strength of modified CF materials apparently increases when the content of PR additive increases from 0 to 9 wt%, relying on the enhancement of bending and stretching of micropores caused by the decrease of average diameter of micropores and the augmentation of the ligament thickness. 

**Figure 6:**
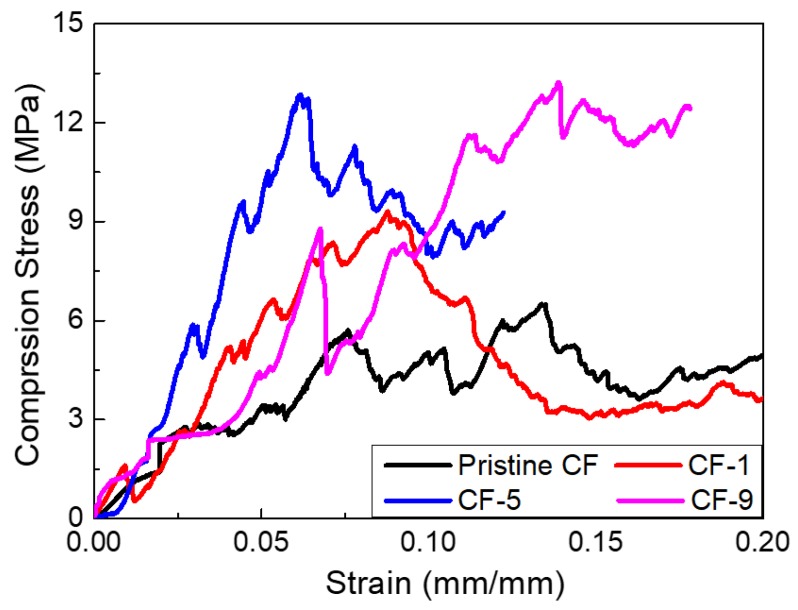
Comparison of compression strength of pristine CF and modified mCF materials with different PR additive.

Figure 6 displays a typical stress-strain graph of pristine CF and mCF-1, mCF-5 and mCF-9. It can be clearly seen that the three stages deformation mechanism exists in all samples, such as the elastic deformation regime, which is a plateau region with plastic deformation and final failure [31]. In the initial stage, the stress-strain curves of CF, mCF-1 and mCF-5 show an enhanced slope and stress when PR content increased from 0 to 5 wt%, verifying that the enhanced compressive strength of CF has been gained. This is probably due to the produced aromatic macromolecules reducing the propagation rate of microcracks under the loading and the compressive strength increasing. With the continue increase of the loading, the stress will reach the yield values, and then elongation occurs under the constant stress and forms plateau regions. This suggests that those samples have a nonrigid deformation with the addition of additive. However, by further increasing the content of additive to 9 wt%, the strength of mCF-9 is almost unchanged compared with the mCF-5, which implied that a slightly excessive PR would not obviously reduce the mechanical performance of CF. 

### 3.6. Thermal Conductivity of Pristine CF and Modified mCF Materials

**Figure 7:**
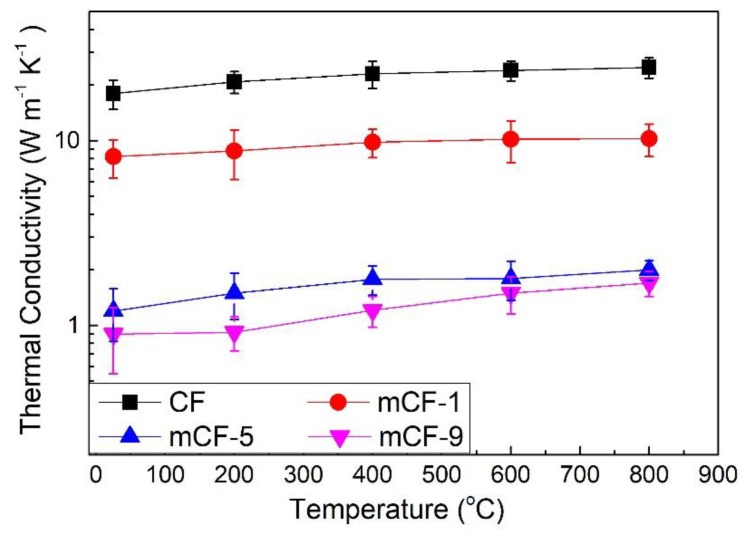
Comparison of thermal conductivity of pristine CF and mCF materials with different PR additive.

Figure 7 illustrates the thermal conductivity (λ) of pristine CF, mCF-1, mCF-5 and mCF-9 materials in the range of temperature from 25 to 800 °C. From Figure 7, the λ of the pristine CF has a maximum value at 18.2 W m^−1^K^−1^, which can be attributed to its graphite-like structure [43,44]. This is consistent with the XRD result. However, when the mass fraction of PR increases from 0 to 5 wt%, the λ of mCF-1 and mCF-5 extraordinarily decreases to 8.2 W m^−1^K^−1^ and 1.2 W m^−1^K^−1^, which is lower 55% and 94.1% than that of pristine CF, respectively. This might be explained by the difference in the crystal structure of pristine and m-CF series materials. For the mCF-1 and mCF-5, due to the increase of disordered non-graphite structure (such as, the 69.8% graphitization degree for mCF-5 and 93.02% for pristine CF), the thermal diffusion rate is substantially influenced, which resulted in λ decreasing. Further increasing the amount of PR to 9 wt%, the λ of mCF-9 decreased to 0.9 m^−1^K^−1^, which is almost same value with mCF-5 and therefore could yield a slightly different crystal lattice structure. Generally, the λ of porous carbon materials has a stronger relationship with their bulk density [44]. Nevertheless, in our case, the λ and bulk density have no obvious relation. Hence, due to the high compressive strength and low λ of this resultant CF, this material may have some potential applications in rocket nozzles, energy saving buildings and other areas.

## 4. Conclusions

In summary, an effective modification method was developed to produce carbon foam (CF) by a co-carbonization process of raw CTP with PR in the presence of catalyst MBSA. Results showed that the crystal structure and uniformity of mCF-5 were dramatically approved owing to the balance between the reduction of released volatile component (such as TS) and formation of macromolecular aromatic compounds (such as QI) during the foaming process. On account of the synergistic effect of the enhancement of bending and stretching of micropores and augment of ligament thickness, the σ of modified CF material was substantially augmented. Moreover, the λ of mCF-9 material decreased by 95.1% compared to pristine CF because of the increase of the disordered non-graphite structure of resultant material (corresponding the reduction of degree of graphitization) after the incorporation of PR additives. Therefore, our work provides a simple and reliable modification method for further design of CF with high σ and low λ for practical application in the civil and military fields.

## Figures and Tables

**Table 1:** Solubility class separation of the CTP and modified CTP by sequential Soxhlet extraction.

Samples	TS (wt%) ^a^	QI (wt%) ^b^
CTP	79.2	3.9
CTP-1	77.4	4.4
CTP-3	75.8	5.8
CTP-5	74.8	6.5
CTP-7	75.8	5.6
CTP-9	76.4	5.3

^a^, Toluene Soluble; ^b^, Quinoline Insoluble.

**Table 2:** XRD d-spacing values, graphitization degree and Raman *I*_D_/*I*_G_ ratios of pristine CF and mCF-5, respectively.

Sample ID	XRD *d*_002_ (nm)	Graphitization Degree (%)	Raman (*I*_D_/*I*_G_)
Pristine CF	0.352	93.02	0.51
mCF-5	0.350	69.80	0.99

λ (Cu Kα) = 0.15406 nm.

**Table 3:** Summaries of bulk density and compressive strength of the pristine CF, mCF-1, mCF-5 and mCF-9.

Samples	Amount of Additive of PR (wt%)	Bulk Density (g/cm^3^)	Compressive Strength (MPa)
Pristine CF	0	0.53 ± 0.1	6.5 ± 0.7
mCF-1	1	0.64 ± 0.1	9.3 ± 1.2
mCF-5	5	0.72 ± 0.1	12.8 ± 0.5
mCF-9	9	0.74 ± 0.1	13.1 ± 0.6
